# Degradable Elastomers: Is There a Future in Tyre Compound Formulation?

**DOI:** 10.3390/molecules26154454

**Published:** 2021-07-23

**Authors:** Marco Naddeo, Gianluca Viscusi, Giuliana Gorrasi, Daniela Pappalardo

**Affiliations:** 1 Prometeon Tyre Group S.r.l., Viale Sarca, 222, 20126 Milano, Italy; marco.naddeo@unisannio.it; 2 Dipartimento di Scienze e Tecnologie, Università del Sannio, Via de Sanctis snc, 82100 Benevento, Italy; 3 Dipartimento di Ingegneria Industriale, Università di Salerno, Via Giovanni Paolo II 132, 84084 Fisciano, Italy; gviscusi@unisa.it (G.V.); ggorrasi@unisa.it (G.G.)

**Keywords:** thermoplastic elastomers, degradable polymers, tyre, block copolymers, polylactides

## Abstract

Problems related to non-biodegradable waste coming from vulcanized rubber represent one of the pre-eminent challenges for modern society. End-of-life tyres are an important source of this typology of waste and the increasingly high accumulation in the environment has contributed over the years to enhance land and water pollution. Moreover, the release into the environment of non-degradable micro-plastics and other chemicals as an effect of tyre abrasion is not negligible. Many solutions are currently applied to reuse end-of-life tyres as a raw material resource, such as pyrolysis, thermo-mechanical or chemical de-vulcanisation, and finally crumbing trough different technologies. An interesting approach to reduce the environmental impact of vulcanised rubber wastes is represented by the use of degradable thermoplastic elastomers (TPEs) in tyre compounds. In this thematic review, after a reviewing fossil fuel-based TPEs, an overview of the promising use of degradable TPEs in compound formulation for the tyre industry is presented. Specifically, after describing the properties of degradable elastomers that are favourable for tyres application in comparison to used ones, the real scenario and future perspectives related to the use of degradable polymers for new tyre compounds will be realized.

## 1. Introduction

A tyre is constituted by polymers, inorganic fillers, chemicals, and reinforcing fibers (Table 1) [1]. Since the last century, the tyre industry has focused on the development of new materials and technologies with the purpose to guarantee safety and handling to the final users in different kinds of applications of tyres, such as passenger cars, trucks and buses, motorcycles, etc. During the last 10 years, the future of tyre technology has observed new horizons, looking not only at performances that are related to safety parameters, such as dry and wet grip, but also at environmental aspects. The European Regulation n° 1222 of 2009 established that all tyres produced from November 2012 must be provided to consumers with all the information on the safety (wet grip index), fuel efficiency (rolling resistance index), and noise in the use condition (external rolling noise index) [2]. Both the rolling resistance and noise indexes are strictly related to CO_2_ emission, intended as energy dissipation for electric vehicles, and noise pollution, respectively [3,4]. The introduction of parameters that take into account environmental aspects has marked an important change in the mind-set of the tyre industry about the development of new products. Despite the high attention on these aspects, there are still other environmental issues related to the use of the actual tyres that should be considered. The most important issue is represented by the end of life tyre (ELT). When a tyre ends its life, the used materials cannot be completely recyclable but turned into “second-life” materials [5,6]. This characteristic is strictly due to the irreversible vulcanization process of the unsaturated elastomeric matrix in which different kinds of chemical products, such as metal oxides, accelerant, antioxidants, resins, and oils, are dispersed. In many cases, ELT are collected in huge landfills or dispersed in the environment, generating social and health problems [7]. In Europe, as reported by the European Tyre and Rubber Manufacturers Association (ETRMA), almost 48% of ELT produced per year is burnt for energy recovery, while 52% is recovered and reused in different fields, such as civil engineering, or again for rubber compounding [8]. Another problem is related to the release into the environment of wear and tear micro-particles coming from the friction and shear between the elastomeric compound of the tread and the road (tyre and road wear particles, TRWPs) [9]. In Europe, around 1.3 million tons of tyre wear are generated every year [10]. Considering that the tyre tread compounds of on-road and off-the road (OTR) vehicles are composed of more than 50% of vulcanized natural rubber and/or synthetic rubber (butadiene rubber, and styrene-butadiene rubber), the polymeric matrix plays a crucial role when TRWPs are dispersed into the environment [11]. Indeed, the degradation products coming from most tread compounds cannot be bio-degraded from the environment, thus accumulating in water and soils [12,13]. For example, synthetic polybutadiene and styrene-butadiene copolymeric rubbers undergo degradation mostly by the photo-oxidation mechanism, and *cis*-1,4-polybutadiene is resistant towards biotic and abiotic oxidation processes [14,15]. Only *cis*-1,4-polyisoprene rubber seems to be more prone to degradation [15].

In general, the biodegradation of tyre wear is hampered by the vulcanization process and the presence of toxic additives and Zn in the tyre tread formulation [19]. Progress in biobased rubber has been reported [20,21,22]. Moreover, interesting research has been carried out on rubber desulfurization [23,24,25,26].

Many studies have recently been carried out to understand the effect of particles from tyre wear when they are dispersed in soil and fresh and seawater [10], and the consequence of the dispersion of these materials into the environment is under evaluation by the European TRWP platform. It is not excluded that the European Regulation will also include an abrasion index to classify the next generation of tyres. Due to the environmental issue, many tyre companies are trying to avoid the use of non-renewable materials and non-biodegradable polymers to move toward greener and eco-friendly solutions [27].

**Figure 1:**
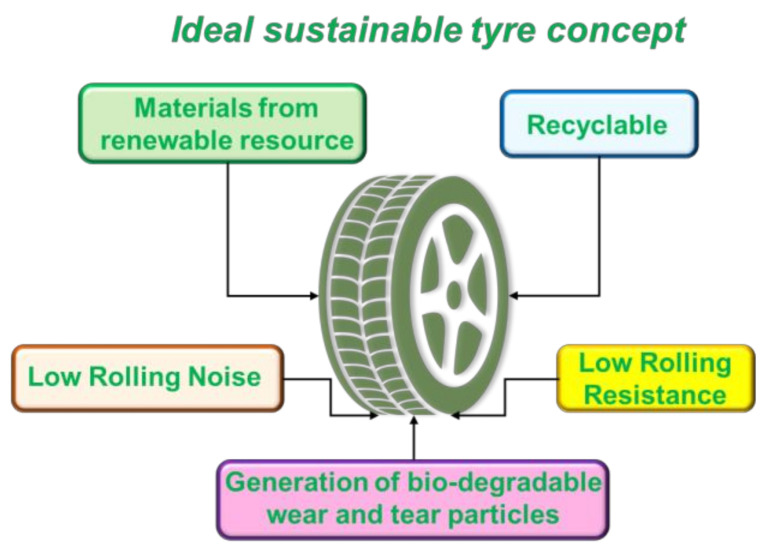
Proposal of an ideal sustainable tyre concept.

The ideal sustainable tyre should be recyclable, composed of material from renewable resource (polymers, chemicals, and fillers), and have a low rolling resistance index (low CO_2_ consumption, or low energy dissipation) and low rolling noise index (reduced noise pollution). Finally, it should generate bio-degradable wear and tear micro-particles under use conditions. This sustainable tyre concept (Figure 1) implies thinking “out of the box” to overcome the problems generated by the current family of materials used for tyre formulation.

### 1.1. Thermoplastic Elastomers

Following this concept, a first approach to the sustainability and recyclability of elastomers from tyre compounds could be represented by the use of degradable thermoplastic elastomers (TPEs). The characteristics of thermoplastic elastomers are summarized in the following paragraph.

#### 1.1.1. The General Structure of Thermoplastic Elastomers (TPEs)

**Figure 2:**
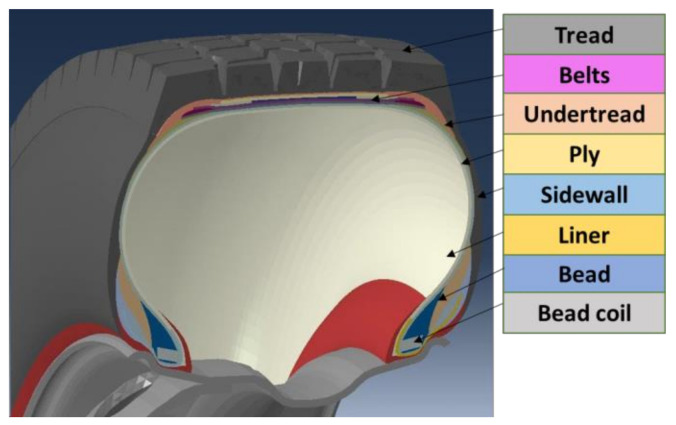
Typical internal structure of a truck tyre.

The elastomeric matrix represents around the 60–65% of the compounds of a tyre structure. Generally, a tyre consists in different compounds assembled with each other to form a complex architecture as shown in Figure 2, where the general structure of a truck tyre is described [28].

Each compound plays a crucial role in terms of tyre performance, integrity, and safety. For example, the tread is important because it is the only compound in contact with the road, and it gives a relevant impact on performances, such as the wet grip and fuel efficiency. This formulation has also to be resistant to tear and abrasion. Furthermore, the rubber compound dedicated to the belts is responsible for the tyre integrity and durability. Then, the liner compound has a primary function in maintaining impermeability in order to avoid rubber oxidation at high temperatures [28]. Considering the different and specific characteristics required by each compound, a precise class of elastomers is commonly used to meet these needs. Table 2 shows the most used elastomeric polymers in the tyre industry associated with the main feature of the compounds [29].

The polymers summarised in Table 2 are unsaturated elastomers, which are subjected to a vulcanization process promoted by sulphur or peroxides. The vulcanisation reaction regards the formation of chemical crosslinks between the C=C double bonds [30], leading to a three-dimensional network, which significantly improve the elastic feature and mechanical properties of the native elastomers, as well as the chemical resistance and thermal stability. After vulcanization, it is not possible to bring back the polymers to their initial state; therefore, this reaction means a vulcanized tyre is not recyclable. Although overcoming the vulcanization process represents a very challenging commitment, the replacement of cross-linked rubber with thermoplastic elastomers (TPEs) is very promising for the recyclability of the tyre. 

According to the ISO 18064, a thermoplastic elastomer is defined as a “polymer or blend of polymers that has properties at its service temperature similar to those of vulcanized rubber but can be processed and reprocessed at elevated temperature like a thermoplastic” [31]. The presence of physical three-dimensional networks allows rubber elasticity to be exhibited over a specified service temperature range, but at the same time, it confers the possibility to process the materials as a thermoplastic at elevated temperatures [28,32].

**Figure 3:**
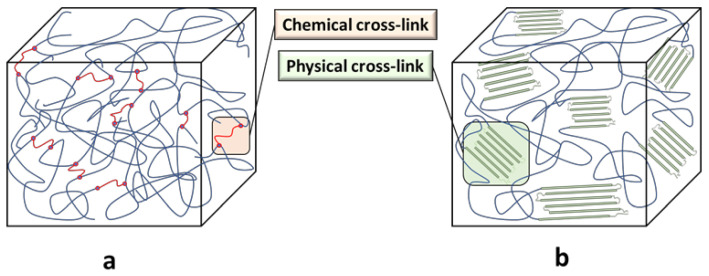
Chemical cross-linked elastomer (**a**) and physical networks in TPEs structure (**b**).

The nature of TPEs consists in two thermo-reversible polymeric phases, which replace the chemical crosslinks that are a result of the vulcanization of thermoset elastomers (Figure 3) [33]. The coexistence of two different phases is crucial to confer to TPEs properties like elasticity and tensile strength. The phase separation is promoted by the chemical architecture of the elastomeric polymers, which are block copolymers with di-block (AB), tri-block (ABA), or multiblock structures [32,34]. To guarantee the formation of the physical crosslinks, the polymeric blocks have two opposite types of structure called “hard” and “soft” blocks. In particular, the hard block is the main component of the physical crosslinks (Figure 3) and it is responsible for the strength of TPEs. This phase is usually obtained by the formation of small crystallites that, in general, are not permanent and may disappear under swelling or with an increase of the temperature. Indeed, the melting temperature (T*_m_*) and the glass transition temperature (T*_g_*) of the crystallites of the rigid blocks determine the upper application temperature of TPEs. Conversely, the soft blocks have an elastomeric feature characterized by a T*_g_* lower than the application temperature [32].

The phase separation of hard and soft blocks is the consequence of the high incompatibility of these two types of polymer chains, which do not get wet and are not so mutually soluble as to lead to a homogeneous phase [32,35].

The advantages of the TPEs, compared to cross-linked rubbers, are the processability and recycling by using the injection moulding or extrusion methods, already used for thermoplastic materials [32,35].

The elastic feature of TPEs, associated with the thermo-reversible properties of the physical networks, opens the possibility to develop recyclable compounds for tyre applications [32]. In this regard, several patents have been published by Michelin, where the use of commercial thermoplastic elastomers based on styrene-butadiene or styrene-isoprene block copolymers has been reported for tread formulations [36,37,38,39]. Remarkably, all TPEs reported in the patents are based on monomers from non-renewable resource and lead to non-degradable or biodegradable polymers.

The application and use of degradable thermoplastic elastomer in tyre formulation still represent a distant goal for the tyre industry. On the contrary, the academic world has begun to work toward the development of degradable TPEs. In the literature, there are brilliant examples of degradable TPEs obtained with different chemical processes and also from renewable resources. Within this thematic review, we wish to give an overview on the degradable elastomers, also derived from renewable resources, that could have potential in compound formulation for the tyre industry. Specifically, by describing the properties of degradable elastomers that are promising for tyre applications, such as those proposed by Michelin, the real scenario and future perspectives related to the use of degradable polymers for new tyre compounds will be presented. In particular, this review will focus on degradable polymers that have similar features to the non-degradable commercially available ones, and therefore they show promising features in terms of physical-mechanical properties for the tyre industries.

#### 1.1.2. A Glance at Thermoplastic Elastomers Proposed by Michelin: Features and Mechanical Properties Required for Tyre Compounds

The commercial thermoplastic elastomers (TPEs) for tread formulations, reported by Michelin, are based on styrene-butadiene or styrene-isoprene block copolymers. Interestingly, the wet grip performances of a tread compound composed of only polystyrene, polyisoprene, and polystyrene (SIS) linear triblock copolymer (Hybrar 5125 ^TM^ from Kuraray) showed an improvement of 13% in the wet grip performance in comparison with a traditional tread compound based on silica-filled SBR [36].

Moreover, an improvement regarding the tear resistance was reported to occur by the partial substitution of natural rubber (NR) with the thermoplastic elastomers SIS polymer Kraton™ D1161 and polystyrene, polybutadiene, polyisoprene, and polystyrene (SBIS) linear triblock copolymers (Kraton™ D1170) in tread formulation [37].

The use in tread formulation of fully hydrogenated (S.O.E.™ L606 by Asahi Kasei) and partially hydrogenated (S.O.E.™ S1611 by Asahi Kasei) polystyrene thermoplastic elastomer (SEBS) leads to an improvement of the dry grip and rolling resistance performances [38].

Finally, different formulations based only on linear triblock copolymers Styrene Butadiene Styrene (SBS) (Europrene^®^ Sol t 166 by Versalis) and Styrene Isoprene Styrene (SIS) (Hybrar 5125 ^TM^ from Kuraray and Kraton™ D1161) could be improved in terms of their mechanical properties with temperature [39].

**Figure 4:**
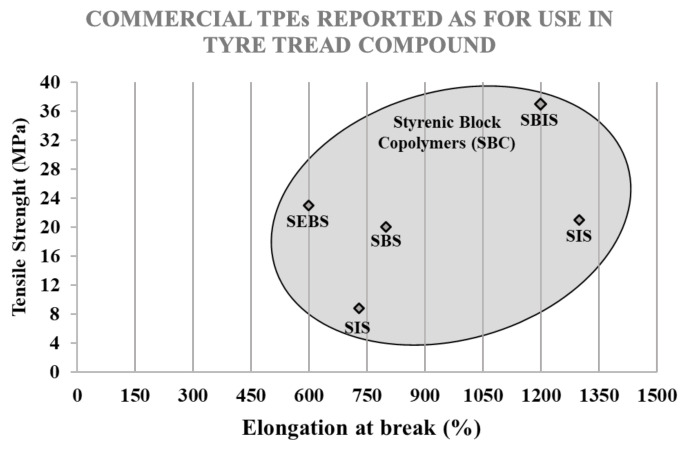
Relation between the tensile strength and the elongation at break of the TPEs reported in Table 3.

The attention is on the characteristic and mechanical properties of this class of TPEs, due to their optimal performances in tyre formulation. To this end, the main properties of the TPEs suitable for tyre tread formulation, described by Michelin, are reported in Table 3. The relation between the tensile strength and the elongation at break of each copolymer is reported in Figure 4. On this basis, the examples described in the literature of degradable TPEs, obtained from renewable resources, with similar or comparable physical and mechanical properties to those of the Michelin TPEs were analysed. The aim of the study was to detect, in the basket of degradable TPEs, suitable candidates that, for mechanical and thermal properties, have the potential to replace the traditional Michelin TPEs. With this scheme, a model of ideal sustainable tyre is going to be proposed. According to this model, in this review, degradable TPEs with mechanical features in line with the Michelin standard were selected

#### 1.1.3. Review Structure

The review is organized in sections. After a first glance on the Michelin-based TPE, the degradable TPEs are classified on the basis of the nature of rigid and of the soft blocks. Most of the degradable TPEs are based on aliphatic polyesters, and have polylactide (PLA) as a rigid block. The classification was made on the basis of their composition. In each section, the chemical features and the thermal and mechanical properties are discussed.

## 2. Copolymers Having Poly(lactide) as a Rigid Block 

Considering the chemical structure of the rigid block based on PLA (as PLLA or PDLA or stereocomplex), the classification of the copolymers has been based on the different structures of the soft blocks. Copolymers with various types of soft block have been described, such as poly(isoprene), different aliphatic polyesters derived from renewable resources, and copolymer of caprolactone and lactide.

### 2.1. Copolymers Having Poly(isoprene) as Soft Block

Frick et al. [40] reported the preparation of polylactide-*b*-polyisoprene-*b*-polylactide (PLA-PI-PLA) triblock copolymers with different compositions and morphologies. The ¦Á,ω-dihydroxy polyisoprene (HO-PI-OH) was used as an initiator; the ring-opening polymerization of lactide was carried out in the presence of Al(O-i-Pr)_3_ catalyst. The reaction was controlled by controlling the Al to HO-PI-OH ratio, avoiding gel formation. The triblock copolymers were prepared in three different morphologies: (i) lamellar, (ii) spherical, and (iii) cylindrical. Such morphological organization was detected using small-angle X-ray scattering (SAXS) and transmission electron microscopy (TEM). Table 4 reports the morphology and structural data of the samples.

**Figure 5:**
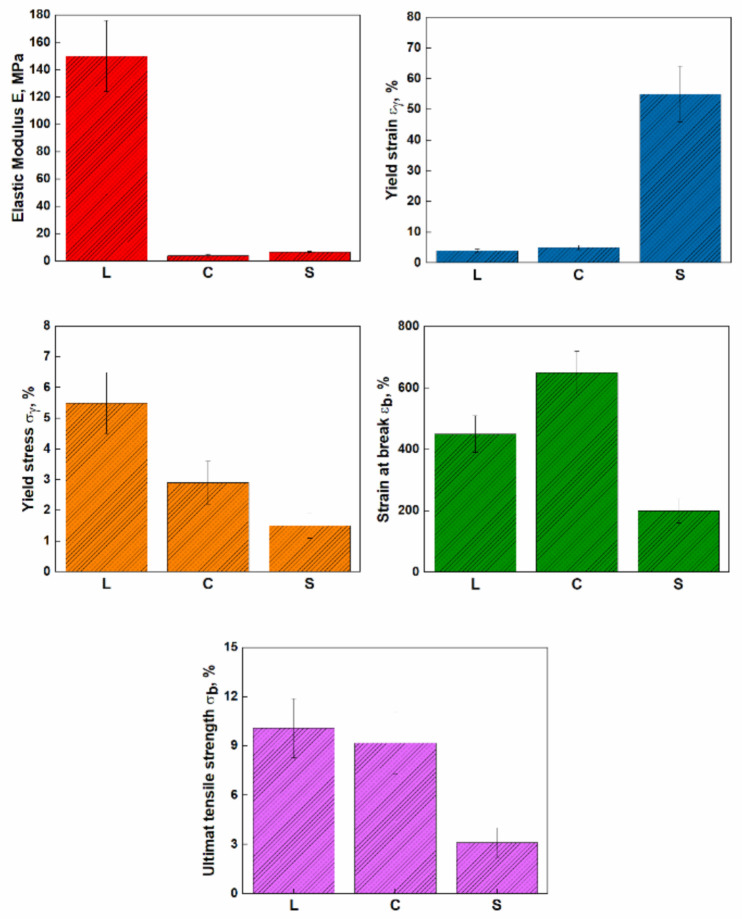
Mechanical properties of PLA-PI-PLA with different morphologies (L = lamellae; C = cylinders; S = spheres). Data from [34].

The mechanical properties (Figure 5) were dependent on the morphology of the materials. Very good strain at break was achieved for samples with cylindrical morphology, showing the best elastomeric recovery.

### 2.2. Copolymers Having Aliphatic Polyesters as Soft Block

**Scheme 1:**
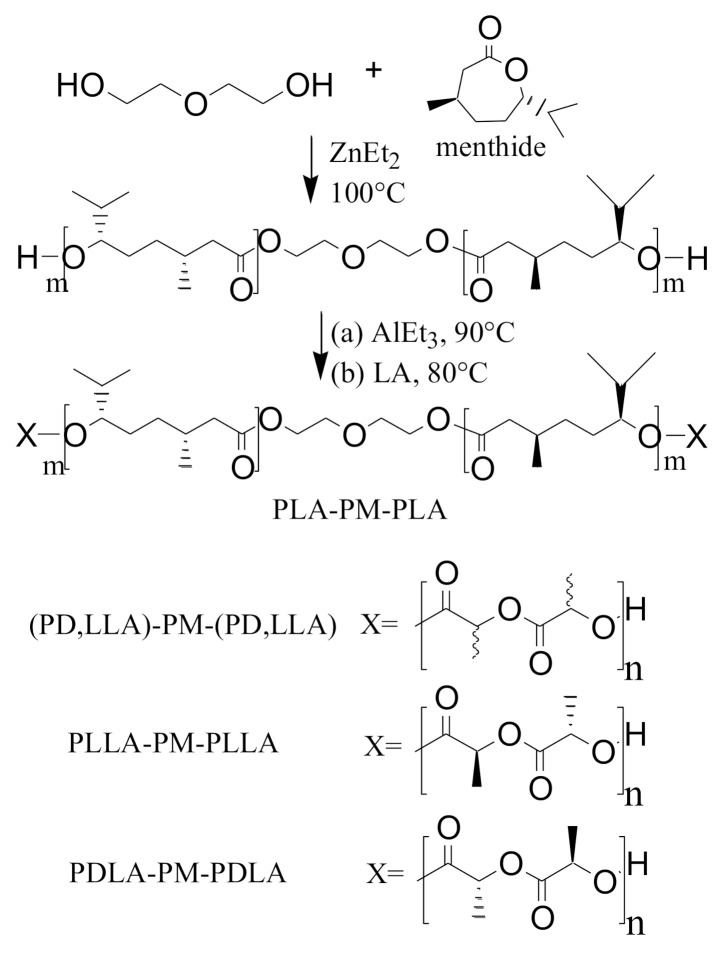
Synthesis of PLA-PM-PLA with various stereochemistry of the PLA blocks. Adapted from [35].

Wanamaker et al. reported the preparation of renewable thermoplastic elastomers, based on polymenthide (PM) as the central block and polylactide (PLA) with various stereochemistry [41]. The properties were varied by modulating the stereochemistry of the end blocks of polylactide (Scheme 1).

The mechanical properties (i.e., elastic moduli and ultimate tensile strengths) of the semicrystalline triblock copolymers were two and three orders of magnitude higher than their amorphous analogues.

Two different kinds of materials, composed of symmetric (50:50) and asymmetric (95:5) blends of triblock copolymers based on two enantiomeric forms of PLA segments forming stereocomplex crystallites, were also prepared. Such blends, compared to the enantiopure analogues, showed significantly higher elastic moduli and similar tensile strengths and ultimate elongation at break.

**Scheme 2:**
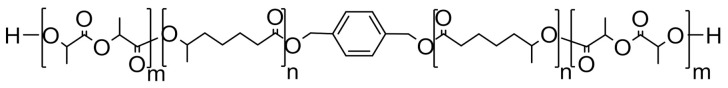
Structure of polylactide-poly(6-methyl-ε-caprolactone)-polylactide, adapted from [36].

Martello et al. [42] reported the preparation of aliphatic thermoplastic amorphous elastomers, polylactide-poly(6-methyl-ε-caprolactone)-polylactide from renewable sources, through sequential ring-opening transesterification polymerization (ROTEP) reaction, as described in Scheme 2.

The Flory–Huggins interaction parameter was determined for two symmetric triblocks from order-to-disorder transition temperatures. The T*_g_* was lower than the one of the equivalent homopolymers. The mechanical properties suggested that the prepared materials possessed elastomeric properties with significant elongation and tensile strength.

Xiong et al. [43] reported the production of a rubbery bio-based and biodegradable polymer with low T*_g_* from β-methyl-δ-valerolactone (βMδVL) and lactide. Block copolymerization of lactide and βMδVL allowed the production of a new class of bio-based and high-performance PLA–PβMδVL–PLA triblock copolymers with tuneable mechanical properties. The possibility of producing a branched lactone, βMδVL, with a high yield from mevalonate by a biosynthetic approach, and the controlled polymerization of this monomer for the production of the PLA–PβMδVL–PLA triblock copolymers was demonstrated. The thermal and mechanical properties of these materials were tuned by controlling the molar mass, architecture, and end block tacticity. The produced materials showed mechanical properties comparable to the commercially available styrenic block polymers.

Watts et al. [44] reported the synthesis of elastomeric thermoplastic block polymers made of poly(γ-methyl-ε-caprolactone) (PγMCL) as the midblock and polylactide (PLA) as the end block, which had an elastomeric nature thanks to physical instead of chemical crosslinks. The incorporation of PγMCL as the midblock with polylactide (PLA) end blocks generated TPEs with high stresses and elongations at break (σ_b_ = 24 ± 2 MPa and ε_b_ = 1029 ± 20%, respectively) and low levels of hysteresis. The use of isotactic PLA as the end blocks increased the strength and toughness of the material (σ_b_ = 30 ± 4 MPa, ε_b_ = 988 ± 30%), due to its semicrystalline structure. It was demonstrated that the exceptional properties of such sustainable materials are due to the entanglements, glass transition temperature, segment–segment interaction parameter, and crystallinity degree. These properties are comparable to the commercial styrene-based thermoplastic elastomers.

### 2.3. Copolymers Having Caprolactone-Lactide Copolymers as Soft Block

Nakayama et al. [45] prepared triblock copolymers made of ε-caprolactone (CL) and lactide. The central block was prepared by the statistical copolymerization of CL with racemic lactide (D,LLA) (30 mol%) using Sn(Oct)_2_ as the catalyst and diethylene glycol as the initiator. This process was followed by the polymerization of L,LA producing PLLA-block-P(CL-stat-D,LLA)-block-PLLA. Such materials showed a very high elongation at break (i.e., 2800%). Triblock polymers with D,LA were also prepared, PDLA-block-P(CL-stat-D,LLA)-block-PDLA. By mixing the L-triblock copolymers with the D-triblocks, blend samples containing stereocomplex crystals were obtained, which showed higher melting points, elastic modulus, and strength compared with those of the homochiral samples.

### 2.4. Copolymers Based on ε-Decalactone Monomer

Lee et al. [46] reported the preparation of adhesive pressure-sensitive TPES, using rosin ester tackifier and elastomers derived from plants. Epoxidized soybean oil was chosen as the plasticizer. The authors produced poly(L-lactide)-poly(ε-decalactone)-poly(L-lactide) (PLLA-PDL-PLLA) block polyesters through bulk ring-opening transesterification polymerization of L-lactide and ε-decalactone (DL) using diethylene glycol as an initiator. Three types of semicrystalline poly(L-lactide)-poly(ε-decalactone)-poly(L-lactide) (PLLA-PDL-PLLA) triblock copolymers were prepared, having semicrystalline hard blocks with molar masses of 10, 20, and 40 kg mol^−1^. The mechanical properties of the triblock architectures were investigated by static and dynamic mechanical analysis. The elastomeric behaviour was found to be dependent on the molar mass composition or amorphous/semicrystalline structure of the end blocks in the triblocks copolymers.

**Figure 6:**
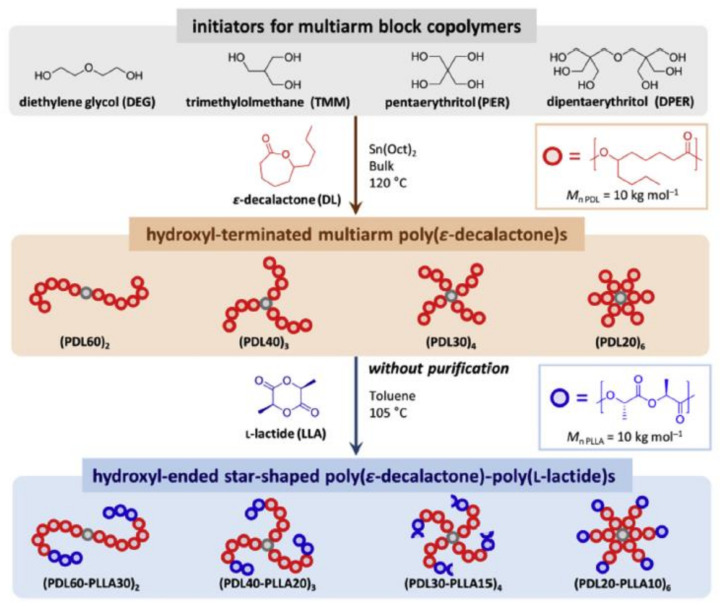
Multiarm block copolymer of ε-decalactone and lactide (from [48] with the permission of *Polymer*).

Lee et al. [47] reported the preparation of a series of well-defined multiarm star block copolymers derived from plant-based monomers. ε-decalactone (DL) with multiarm initiators produced hydroxyl-terminated materials (PDL-OH)_n_ through controlled bulk *ring-opening polymerization*. Such materials were subsequently converted to (PDL-PLLA)_n_ using L-lactide (LLA) through a one-pot two-step process (Figure 6). Structural analysis showed that PLLA hard domains were thinner, and thus, more compact microphase-separated structures with hexagonally packed cylinders were induced by increasing n. The mechanical and thermal properties of the star blocks were evaluated. The thermal degradation stability of the star-shaped thermoplastic elastomers were improved due to the shorter length of the PLLA chain compared to the relatively low T*_d_* of the corresponding linear block. Dynamic mechanical analyses showed that the 3, 4, 6-arm star block PDL-PLLA copolymers retained viscoelastic properties until a temperature of 240 °C. Tensile tests showed that these materials had an elastomeric behaviour; furthermore, their elastic moduli could be modified to values for desired applications by changing the multiarm initiator. Finally, the tensile strength was found to be dependent on the number of PDL-PLLA chains and the elastic recovery remained at good levels with increasing n. Such novel materials can be considered as potential materials for applications where high-performance thermoplastic elastomers are desired.

## 3. Copolymers Having a Rigid Block Other Than Polylactide

Although largely exploited, the use of PLA is restricted by its quite low T*_g_* (55 °C), which confines the thermoplastic elastomers’ upper use temperature, and by its sub-optimal mechanical properties. Notably the effect of the temperature and the T*g* on the mechanical properties of the considered materials are crucial. The importance of hard blocks in PLA copolymers, for instance, is fundamental for increasing the Tg of the final material and confers the manufactures better mechanical properties for temperatures higher than ambient temperature.

While copolymers having a poly(lactide)-based rigid block have been mainly described as thermoplastic elastomers, few examples have been reported in the literature relative to copolymers having a rigid block of a different composition. In the following paragraphs, the structure and properties of copolymers having a rigid block other than PLA will be described. In particular, the focus will be on copolymers with the tulipanin A monomer, semi-aromatic polyesters, and poly(hexamethylene 2,5-furanodicarboxylate) as the rigid block.

### 3.1. Rigid Block Based on Tulipanin a Monomer

Hillmayer et al. [48] designed and synthesized ABA triblock copolymers derived from two plant-based monomers, menthide (M) and methylene-γ-butyrolactone (MBL) or tulipalin A, a natural substance found in the common tulip, by sequential polymerization.

**Scheme 3:**
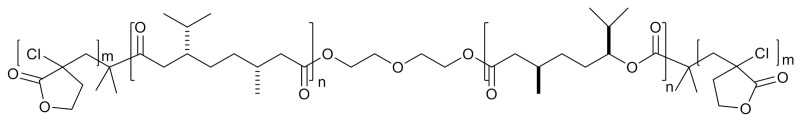
Poly(α-methylene-γ-butyrolactone)-*b*-poly(menthide)-*b*-poly(α-methylene-γ-butyrolactone) (PMBL-PM-PMBL).

The central soft block was constituted by hydroxy terminated polymenthide (PM); it was obtained through controlled ring-opening transesterification polymerization of menthide with Sn(Oct)_2_. Subsequently, the dibromo end-functionalized poly(menthide) was prepared by an esterification reaction, using 2-bromoisobutyryl bromide, and the desired PMBL-PM-PMBL triblock copolymers were synthesized using copper-catalysed atom transfer radical polymerization of MBL (Scheme 3).

Four triblock copolymers with different compositions were prepared; their architectures were confirmed by NMR, while SEC proved narrow molar mass distributions. The PM and PMBL blocks were expected to be incompatible, due to the polar nature of PMBL and the relatively nonpolar nature of PM. Accordingly, phase separation was evidenced by DSC, AFM, and SAXS. Tensile experiments showed excellent elongation, strain, and elastic recovery properties for the PMBL-PM-PMBL triblock copolymers.

In detail, PMBL-PMPMBL (9−100−9) gave a Young modulus comparable to that of commercial poly(styrene)-poly(butadiene)-poly(styrene) (SBS) TPEs (>6.0 MPa) [49]. The high Tg of PMBL (195 °C) [50] resulted in thermoplastic elastomers that retained their mechanical behaviour at elevated temperatures.

### 3.2. Semi-Aromatic Polyester as Rigid Block

**Scheme 4:**
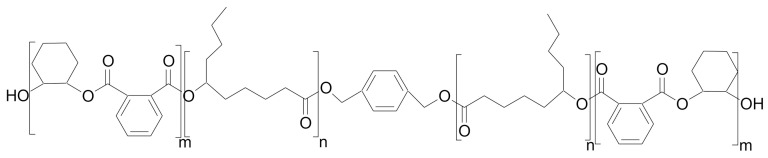
Structure of poly(cyclohexene-*alt*-phthalate)-*b*-poly(3-decalactone)-*b*-poly(cyclohexene-*alt*-phthalate).

Williams et al. [51] developed ABA triblock copolymers having a soft block made of poly(ε-decalactone) and rigid B block made of semi-aromatic polyesters. The copolymers were prepared by sequential monomers’ addition in the presence of a Zn/Mg-based dinuclear catalyst. The central inner soft block was obtained by polymerization of ε-decalactone, a monomer derived from castor oil, while the subsequent addition of phthalic anhydride (PA) with cyclohexene oxide (CHO), by a controlled alternating ring-opening copolymerization (ROCOP), afforded the outer B blocks (Scheme 4). The ROCOP of PA and CHO can afford a semiaromatic polyester with a Tg from 133–146 °C, depending on the molar mass [52].

Two series of samples with different compositions and molar masses were synthesized. For copolymers with volume fractions of the hard block (*f*_hard_) of 0.4, block immiscibility was indicated by the two glass transitions in the DSC analysis, close to the values expected for the constituent hard and soft blocks.

These triblock polyesters showed tensile mechanical properties similar to those of well-known styrenic block polymers, in particular to polystyrene-*b*-polyisoprene-*b*-polystyrene (SIS). Very interestingly, they showed greater elongations at break, a wider operating temperature window, and a higher upper service temperature than SIS. Furthermore, at variance with SIS, they could be degraded by hydrolysis using organic acids or by treatment with the lipase Novozyme.

### 3.3. Poly(hexamethylene 2,5-furanodicarboxylate) as Rigid Block

**Scheme 5:**
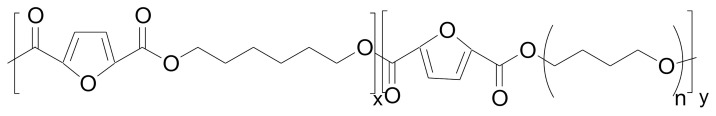
Structure of poly(hexamethylene 2,5-furanodicarboxylate)-*b*-poly(tetrahydrofuran).

Goracy et al. prepared a series of poly(ether-ester)s copolymers using monomers derived from renewable resources by a two-stage procedure, consisting of transesterification and polycondensation processes in the presence of Ti(OBu)_4_ [53]. The multiblocks copolymers were constituted by a hard block made of poly(hexamethylene 2,5-furanodicarboxylate) (FHD) and poly(tetrahydrofuran) (F-pTHF) soft sequences (Scheme 5). The copolymers were prepared with different compositions (from 75 to 25 in mol of the two monomers). The copolymers were fully characterized by FTIR, NMR, DSC, DMTA, and X-ray diffraction analyses.

The T*_m_* attributed to the PHF was variable with the composition (102–146 °C). For all the copolymers prepared, only one T*_g_* was observed, with a value variable with the composition (−66–16 °C).

The characterization obtained by DSC and DMTA analyses, along with SEM observations, disclosed the phase structure of PHF-*b*-F-pTHF copolymers. In particular, in the obtained copolymers, the hetero-phase structure resulting from semicrystalline and amorphous domains was evident. The mechanical tests showed that the tensile strength of the copolymers decreased with the decrease of the crystallization segments. The elongation at break, instead, increased by increasing the amorphous segment.

In the series of synthetized copolymers, it was found that the PHF-*b*-F-pTHF copolymer, characterized by a composition of PHF: F-pTHF of 25:75, resulted in a promising shape memory polymer, with a shape fixity of over 90% and shape recovery efficiency of over 60%.

## 4. Conclusions and Perspective

In this review, a systematic report of degradable thermoplastic elastomers (TPEs) derived from renewable resources is presented. The focus of the survey was on degradable TPEs having thermal and mechanical properties close to those of commercial non-degradable TPE proposed by Michelin.

Here, a general consideration of the term “degradation” is mandatory. The term degradation includes a wide range of chemical and physical phenomena.

In the case of tyres, the first phenomenon of degradation, called “abrasion*”*, is the generation of micro-particles as a consequence of the contact of the tyre-tread compound and the road. In this sense, the comparison of the abrasion resistance of synthetic thermoplastic elastomers (SBS, SIS, etc.) and the biodegradable thermoplastic elastomers proposed in the review is a field of research that is still unexplored.

The second phenomenon called “thermo-oxidative degradation” consists in the radical scission of the polymeric chains and the break of the sulphur bonds under high-temperature and oxygen/ozone conditions. In this sense, poly-dienes like SBR, BR, and NR are very susceptible to this kind of degradation. Indeed, different antioxidants and anti-ozonants are currently used for tyre compounds. This kind of degradation is very critical, and it is due to the presence of the unsaturated double bonds still present after the vulcanisation process.

For most of the biodegradable thermoplastic elastomers proposed in this review, there are no double bonds nor conjugated dienes within the molecular structure of the polymers. This could represent a great advantage in terms of the thermo-oxidative degradation resistance properties, which should avoid, or limit, the use of such protective agents.

In addition to these phenomena, in the case of aliphatic polyesters, other effects must be considered, mainly driven by the hydrolysis of ester bonds. The degradation of aliphatic polyesters has been largely addressed in the literature with enfaces also on the importance of the lifetime of degradable polymers [54,55,56,57,58]. As a result, these aspects were not covered by this review.

It is important to consider that at the moment, the fundamental understanding of degradation mechanisms and interactions, particularly in a natural environment, is still not sufficiently advanced to produce a single unified theory. As a consequence, the degradation of the different materials must be studied case by case. Moreover, a corrected balance between the durability required in technical applications, and the degradability, urgently necessary for the environmental issues needs to be assessed. The scope of this review also encourages new research activities in this field in order to move to more sustainable compounds for tyre applications.

**Figure 7:**
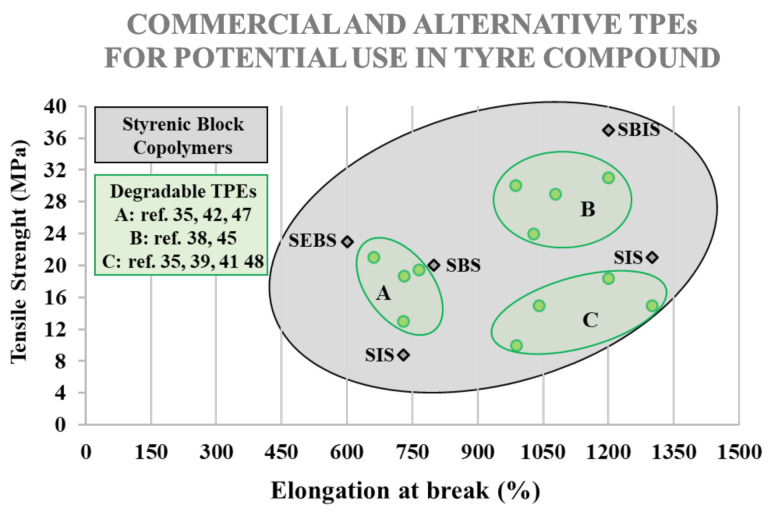
Comparison of the mechanical properties of degradable TPEs with the commercial non-degradable TPEs.

The most representative degradable TPEs, having properties that are promising in tyre tread formulations, are summarized in Table 5. In Figure 7, a comparison of the mechanical properties of degradable TPEs with commercial non-degradable TPEs is shown.

The ambition of the review was indeed to select degradable polymers that could be promising materials for the development of sustainable TPEs of future tyres. Perusal of the literature showed the presence of several degradable TPEs responding to the desired features, having “hard” and “soft” blocks of different compositions. In particular, several copolymers responding to these features exploited the use of polylactide as a hard component, either as an atactic high T*_g_* block, or as an isotactic semi-crystalline block. In this regard, the use of controlled synthetic strategies, in particular the ring-opening polymerization of cyclic esters, allowed the preparation of the desired microstructures and architectures [60,61].

Beside the PLA-based TPEs, recently, novel copolymers with a rigid block made of polymers other than PLA have been developed. In particular copolymers with a rigid block made of the tulipanin A monomer, poly(hexamethylene 2,5-furanodicarboxylate), and semi-aromatic polyesters have been described. In particular, the preparation of these latter materials was carried out by the alternating ring-opening co-polymerization (ROCOP) of cyclic anhydrides with epoxides, a powerful strategy that has recently emerged for the preparation of structurally diverse polyesters [62,63]. Indeed, in 2014, Williams et al. pioneered an innovative catalyst switch process between ROP and ROCOP achieved by chemoselective control for the preparation of diverse block-copolymers, expanding the method to a large assortment of epoxides, anhydrides, and cyclic esters, and thus also producing innovative polymeric structures [64]. The ROCOP approach was investigated by exploiting a catalyst switch process, for the preparation block-copolymers of cyclohexene oxide, phthalic anhydride, and large ring-size lactones [65].

Among the advances being introduced in the automotive industry today, the increasing use of bioplastics in vehicles is one of the more outstanding. With the growing demand for vehicles and, at the same time, growing pressure on sustainability and environmental concerns, bioplastics have found applications in almost all components either for exterior or for interior functions. After the lifecycle of the tires, the disposal of ELTs represents a serious problem for the next decades. The necessity of producing novel biodegradable materials starting from bio-based building blocks is a crucial challenge. A deep knowledge of the synthesis processes is fundamental to determine the physical properties (in particular, mechanical) of the novel materials, which must be as close as possible to the traditional materials now used for tires.

Considering the important role of carbon black and silica as reinforcing fillers, these traditional ingredients must also be substituted with novel bio-based or degradable ones. In this sense, as an example, lignin represents a sustainable alternative reinforcing filler. Lignin is the second most abundant natural resource, and it has several interesting characteristics, such as degradability and good mechanical and chemical properties [66].

It could be envisaged that the automotive industry will never stop growing, but it is continuously under pressure from environmentalists. Degradable polymers, which could also be derived from renewable resources, may represent a sustainable option for tyres, in order to reduce landfill waste and improve efficacies, contributing to a greener and cleaner environment.

## Figures and Tables

**Table 1:** Composition by weight (%) of the tyres employed for on-road and off-the road (OTR) vehicles [16,17,18].

Material	Passenger Car	Truck and Bus	Off the Road (OTR)
Elastomers	47	45	47
Carbon black and silica	22.5	21	22
Metal fabrics	14	23.5	12
Textiles	5.5	1	10
Vulcanization agents	2.5	3	3
Additives	8.5	6.5	6
Estimated weight of new tire (kg)	8.5	65	>100

**Table 2:** Elastomeric polymers used in tyre compound [29].

Elastomer	Main Feature
Natural Rubber (NR)	Tear resistance/Low rolling resistance
Polyisoprene (IR)	Tear resistance
Butadiene Rubber (BR)	Abrasion resistance
Styrene-Butadiene Rubber (SBR)	Skid resistance
Isobutylene Isoprene Rubber (IIR)	Low gas permeability
Halogenated Isobutylene Isoprene Rubber (HIIR)	Low gas permeability

**Table 3:** Properties of the commercial TPEs for tyre tread formulation, as reported in the technical data sheets by the suppliers.

TPE	Commercial Name	Supplier	Rigid Block	Soft Block	M300%	Tensile Strength	Elongation at Break
			Polystyrene Content	EB/I/B			
			(%)		(MPa)	(MPa)	(%)
Styrene and isoprene Vinyl-Rich triblock copolymer (SIS)	Hybrar 5125TM	Kuraray	20	80	2.8	8.8	730
Styrene and isoprene triblock copolymer (SIS)	KRATONTMD1161	Kraton	15	85	0.9	21	1300
Styrene and butadiene triblock copolymer (SBS)	EUROPRENE^®^SOL T 166	Versalis	30	70	2.5	20	800
Styrene, butadiene and isoprene triblock copolymer (SBIS)	KRATONTMD1170	Kraton	19	81	1.4	37	1200
Hydrogenated Styrenic Thermoplastic Elastomer (SEBS)	S.O.E.TM S1611	Asahi Kasei	62	38	4.0	23	600

**Table 4:** PLA-PI-PLA microstructural data.

Triblock (Morphology ^a^)	*M_n_*(PLA)-*M_n_*(PI)-*M_n_*(PLA) ^b^ (g/mol)	*f*_PLA_^c^	PI PDI ^d^	Block PDI ^d^	*D*^e^ (μm)	*Tg*_(PI)_^f^ (K)	*Tg*_(PLA)_^f^ (K)
Lamellae (L)	14000-33000-14000	0.40	108	1.10	0.0045	210.15	333.15
Cylinders (C) of PLA	9000-33000-9000	0.28	1.08	1.12	0.0038	212.15	334.15
Spheres (S) of PLA	5100-35000-5100	0.18	1.07	1.12	0.0029	211.15	325.15

^a^ Determined by SAXS and TEM. ^b^ Calculated from ^1^H-NMR spectroscopy. ^c^ Volume fraction of PLA calculated using ρ_(PLA)_ 1154 mg/cm^3^ and ρ_(PI)_ = 830 mg/cm^3^ at 413.15 K. ^d^ Determined by SEC. ^e^ Principal domain spacing *D* = 2π/q* (SAXS): T = 393.15 K for triblocks C and L, T = 363.15 K for S. ^f^ Determined by DSC.

**Table 5:** Mechanical properties of degradable TPEs described in this review.

Hard Block	Soft Block	Tensile Strength (MPa)	Elongation at Break (%)	Reference
P(L,L-LA)	Polymenthide	19.5	765	[41]
P(D,L-LA)	Polymenthide	18.7	731	
P(L,L-LA)/P(D,L-LA)	Polymenthide	10.0	990	
P(D,L-LA)	Poly(ε-caprolactone-co-ε-decalactone)	18.4	1200	[59]
P(L,L-LA)	Poly(γ-methyl-ε-caprolactone)	31.0	1200	[44]
P(L,L-LA)	Poly(γ-methyl-ε-caprolactone)	24.0	1029	
P(L,L-LA)	Poly(γ-methyl-ε-caprolactone)	30.0	998	
P(L,L-LA)/P(D,L-LA)	Poly(ε-caprolactone -ran-L,L-LA)/Poly(ε- caprolactone -ran-D,L-lactide)	15.0	1300	[45]
[P(L,L-LA)]_6_	[Poly(ε-decalactone)]_6_	15.2	1041	[47]
Poly(cyclohexene-alt-phthalate)	Poly(ε-decalactone)	29.1	1079	[51]
Poly(hexamethylene 2,5-furanodicarboxylate)	poly(tetrahydrofuran)	21.8	662	[53]
Tulipanin A	Polymenthide	13.0	730	[48]

## Data Availability

Data sharing not applicable.
